# INTER-RELATIONSHIPS OF CIRCULATING PLASMA METABOLITES, INTERMUSCULAR FAT, AND PROCESSING SPEED: HEALTH ABC STUDY

**DOI:** 10.1093/geroni/igae098.3667

**Published:** 2024-12-31

**Authors:** Richard Xu, Megan Marron, Shanshan Yao, Seyoung Kim, Ravi Shah, Venkatesh Murthy, Anne Newman, Caterina Rosano

**Affiliations:** University of Pittsburgh, Pittsburgh, Pennsylvania, United States; University of Pitssburgh, Pittsburgh, Pennsylvania, United States; University of Pittsburgh, Pittsburgh, Pennsylvania, United States; University of Pittsburgh, University of Pittsburgh, Pennsylvania, United States; Vanderbilt University Medical Center, Nashville, Tennessee, United States; University of Michgan, Ann Arbor, Michigan, United States; University of Pittsburgh, University of Pittsburgh, Pennsylvania, United States; University of Pittsburgh, Pittsburgh, Pennsylvania, United States

## Abstract

Higher intermuscular fat (IMF) is associated with worse processing speed, measured by the digit symbol substitution test (DSST) in older adults. However, the underlying biological mechanisms are not well understood. Since muscles and the brain are both highly metabolically active, we sought to identify metabolites that would explain the IMF-DSST association. We assessed 613 known plasma metabolites in 2388 participants from the Health, Aging and Body Composition Study (average age ± standard deviation: 74.7 ± 2.9 years, 50% men, 63% white), using liquid chromatography-mass spectrometry. Using linear regression, we first identified metabolites associated with both IMF and DSST, and then used mediation analyses to assess to what extent these metabolites explained the IMF-DSST association. Higher IMF was associated with worse DSST scores (standardized beta (95% CI): -0.08 (-0.12, -0.03), p< 0.001) adjusting for age, sex, and race. Adjusting for covariates and multiple comparisons, 66 metabolites were associated with both IMF and DSST and 24 of these significantly attenuated the IMF-DSST association. Four attenuated the association by ≥10-14%: higher levels of adrenic acid (omega-6 polyunsaturated fatty acid), and lower levels of maslinic acid, 1-methylnicotinamide (myokine derived from vitamin B3), and C20:5 lysophosphatidylcholine (lysophospholipid containing omega-3 fatty acid) were associated with higher IMF and worse DSST. Prior studies suggested adrenic acid may have pro-inflammatory effects, while the other three metabolites have neuroprotective, anti-inflammatory, and antioxidant effects. This study provides hypothesis-generating evidence that metabolites related to myokines, fatty acids, and phospholipids may partially explain the negative association of intermuscular fat with processing speed.

